# Recent advances of PIWI‐interacting RNA in cardiovascular diseases

**DOI:** 10.1002/ctm2.1770

**Published:** 2024-07-31

**Authors:** Bo Li, Kai Wang, Wei Cheng, Bo Fang, Ying Hui Li, Su Min Yang, Mei Hua Zhang, Yun Hong Wang, Kun Wang

**Affiliations:** ^1^ Key Laboratory of Maternal & Fetal Medicine of National Health Commission of China Shandong Provincial Maternal and Child Health Care Hospital affiliated to Qingdao University Jinan Shandong China; ^2^ Institute for Translational Medicine The Affiliated Hospital of Qingdao University, College of Medicine, Qingdao University Qingdao Shandong China; ^3^ Department of Cardiovascular Surgery Beijing Children's Hospital, Capital Medical University National Center for Children's Health Beijing China; ^4^ Department of Cardiovascular Surgery The Affiliated Hospital of Qingdao University Qingdao Shandong China; ^5^ Hypertension Center Beijing Anzhen Hospital Capital Medical University Beijing China

**Keywords:** biomarkers, CVDs, epigenetics, PIWI‐interacting RNA

## Abstract

**Background:**

The relationship between noncoding RNAs (ncRNAs) and human diseases has been a hot topic of research, but the study of ncRNAs in cardiovascular diseases (CVDs) is still in its infancy. PIWI‐interacting RNA (piRNA), a small ncRNA that binds to the PIWI protein to maintain genome stability by silencing transposons, was widely studied in germ lines and stem cells. In recent years, piRNA has been shown to be involved in key events of multiple CVDs through various epigenetic modifications, revealing the potential value of piRNA as a new biomarker or therapeutic target.

**Conclusion:**

This review explores origin, degradation, function, mechanism and important role of piRNA in CVDs, and the promising therapeutic targets of piRNA were summarized. This review provide a new strategy for the treatment of CVDs and lay a theoretical foundation for future research.

**Key points:**

- piRNA can be used as a potential therapeutic target and biomaker in CVDs.
- piRNA influences apoptosis, inflammation and angiogenesis by regulating epigenetic modificaions.
- Critical knowledge gaps remain in the unifying piRNA nomenclature and PIWI‐independent function.

## Graphical abstract

- 1. piRNA is a class of small noncoding RNA.
- 2. piRNA plays a variety of biological functions in vivo.
- 3. piRNA plays an important role in CVDs.

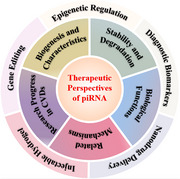

## 1 INTRODUCTION

Cardiovascular diseases (CVDs), globally prevalent, pose a significant risk to health and longevity. Among them, the incidence and prevalence of CVDs in women are higher than that of men, but the mortality is lower than that of men.^1^, ^2^, ^3^ Smoking, alcohol consumption, lack of exercise, diabetes, high blood pressure, hyperlipidaemia and renal insufficiency are the major risk factors associated with death from CVD. Hypertension is the leading risk factor for death from CVDs.^2^ With the increasing prevalence rate, the prevention and control of CVDs is becoming increasingly serious in China.

In the past, researchers have focused on RNA, which codes for proteins, and noncoding RNAs (ncRNAs) have long been considered ‘junk genes’. Recent research highlights the crucial role of ncRNAs in modulating gene expression and cell function. NcRNAs can regulate gene expression in a variety of ways, including miRNA‐mediated targeted gene silencing and lncRNA's influence on chromatin structure and transcriptional regulation.^4^ These studies help us take a deeper understanding of how CVDs regulate various life processes in organisms. Currently, ncRNAs are confirmed to play crucial roles in the important processes of cardiomyocyte proliferation, cardiac remodelling and decompensation, among which delaying ventricular remodelling, reducing cardiomyocyte death and promoting cardiomyocyte regeneration are the research hotspots and difficulties in preventing CVDs.^5^ With further research, we expect that more ncRNA‐related therapies will move from the laboratory to the clinic, bringing new hope to patients with CVDs.

The article not only systematically summarises the biogenesis, degradation, function and mechanism of PIWI‐interacting RNAs (piRNAs), but also emphasises the research progress of piRNAs in the treatment of various CVDs, including myocardial infarction (MI), hypertrophic cardiomyopathy (HCM) and heart failure (HF), etc. The potential of targeting piRNA in the treatment of CVDs and its application as a biomarker of CVDs are discussed. This article provides a comprehensive overview of piRNAs in CVDs research, promotes the progress in the field of ncRNAs research, and provides potential help for the development of new therapeutic strategies for CVDs.

## 2 OVERVIEW OF PIRNA

**FIGURE 1:**
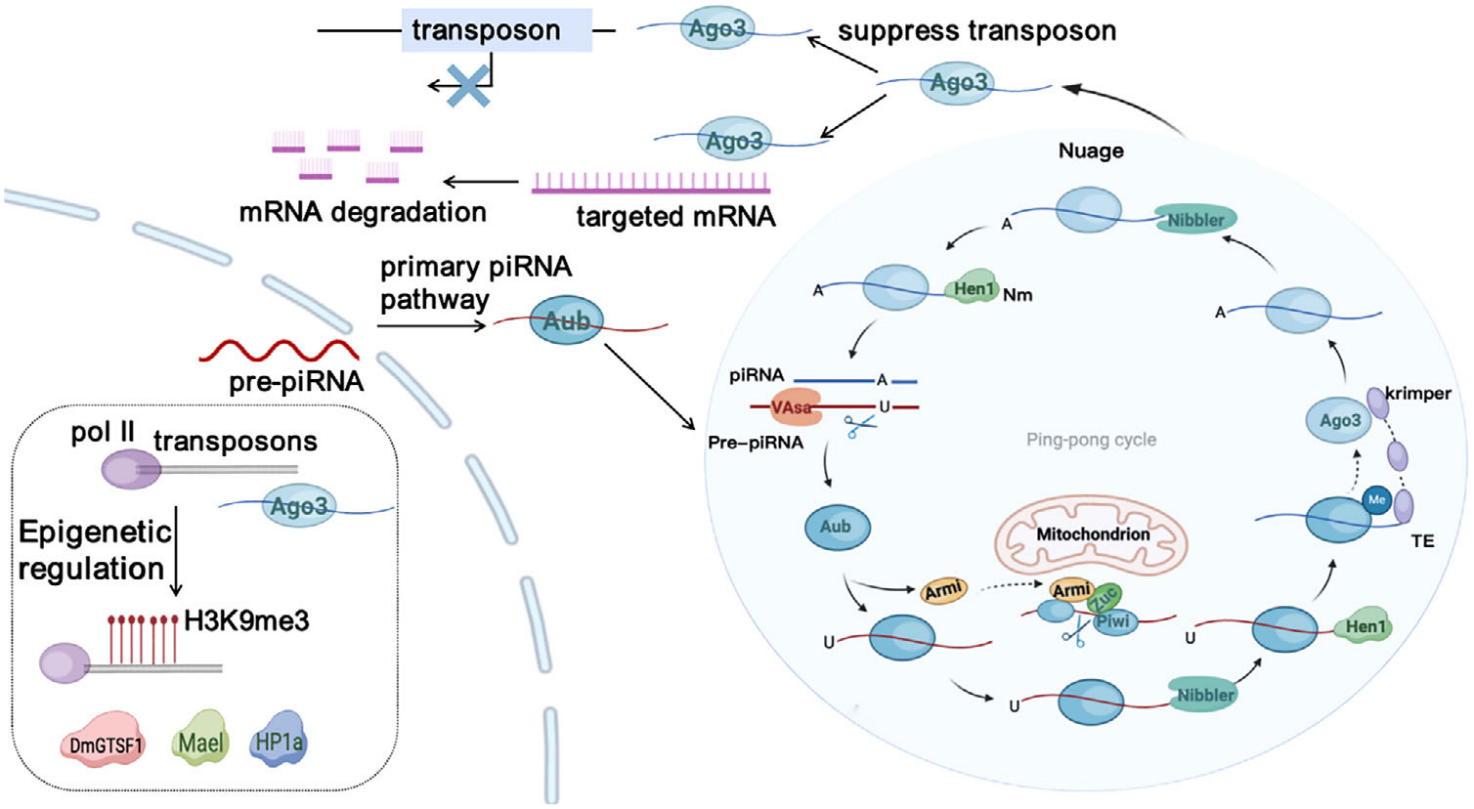
piRNA origin and epigenetic regulation. Pre‐piRNA in the nucleus by primary piRNA pathway into ‘ping‐pong’ circulation generated secondary piRNA, participate in mRNA degradation and transposon targeting inhibition. Meanwhile, factors such as Hen1 and Nibbler are involved. PIWI–piRNA complexes by modified histone marks (H3K9me3) and combined with target genes of Pol II to regulate its target genes, DmGTSF1, Mael, Hp1a play an important role.

piRNA is usually 18−35 nt in length, slightly longer than miRNA and siRNA, with 2′‐O‐methylation (Nm) modification at the 3′ end and a uracil monophosphate nucleotide tendency at the 5′ end. The cleavage of piRNAs differs from other small RNAs in that biogenesis is independent of Dicer and depends on the formation of complexes with PIWI proteins, integral to the Ago family, play crucial roles in germ cell maturation and genome stabilisation.^6^ However, more and more studies have found that piRNAs are widely expressed in somatic tissues and may play an important role outside the germline.^7^, ^8^ The internal ribonucleoprotein particles and the outer mitochondrial membrane are the main production sites of piRNA, and the occurrence process is significantly conserved among different species^9^, ^10^ (Figure 1). piRNA maturation involves the pruning of pre‐piRNA and Nm of its 3′ terminus.^11^ The formation of Nm helps piRNA resist 3′−5′ truncation and 3′‐uridine‐triggered degradation and increases its own stability.^12^ HUA ENHANCER 1 (HEN1) and its homologue are necessary for RNA stability, its lack of expression or mutation and the stagnation of reproductive cells and shorten the service life and neural degeneration, highlighting the Nm in the core role of piRNA function.^13^ In addition, studies have identified ribosomes involved in piRNA biosynthesis.^14^ Research offers fresh perspectives on piRNA synthesis mechanisms. At present, the roles of different piRNAs biosynthetic pathways in the cytoplasm remain unclear, and the roles of sequences, structural motifs, and specific trans‐acting factors in piRNA biogenesis during maturation need to be further explored.^6^, ^15^

**FIGURE 2:**
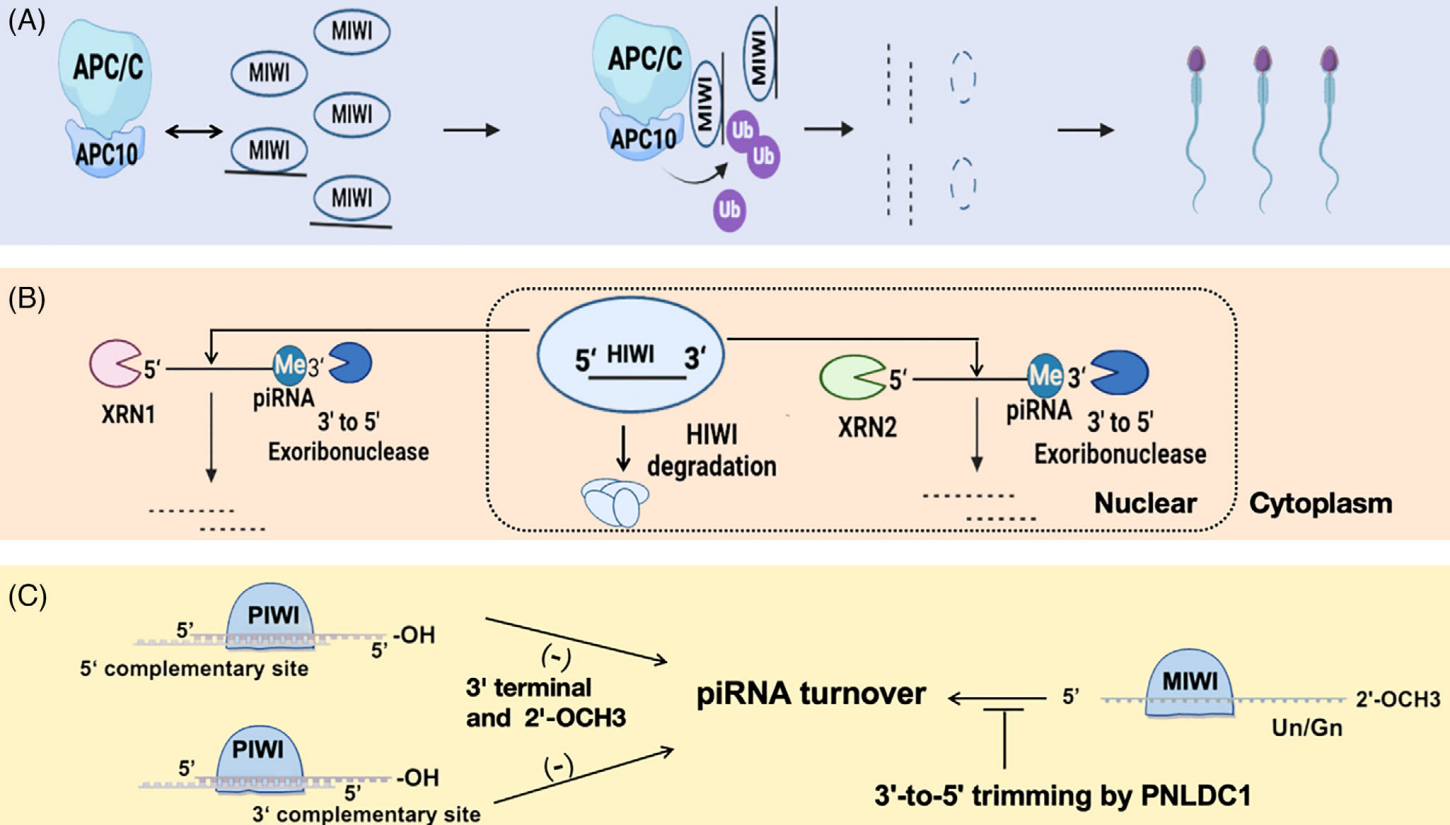
Different mechanisms of piRNA degradation. (A) piRNA loading induces a conformational change of MIWI and MIWI ubiquitination through APC/C, driving the binding of MIWI to APC10 and ultimately degradation through the APC/C‐26S proteasome pathway. (B) 5′ to 3′ exoribonucleases can cause piRNA decay, among which XRN1 is responsible for the degradation of cytoplasmic piRNA, XRN2 is responsible for the degradation of nuclear piRNA, and the 3′ end of piRNA is modified by Nm to reduce the degradation. (C) Trigger sites complementary to 5′ or 3′ sequence of unmethylated piRNAs elicit their decay by distinct mechanisms.

In late spermatids, piRNA degradation depends on a feed‐forward mechanism. Specifically, piRNAs trigger ubiquitination degradation of MIWI, which leads to piRNA clearance.^16^ piRNA enhances MIWI binding to APC/C substrates, promoting degradation via the APC/C‐26S proteasome route (Figure 2A). piRNA degradation primarily occurs through the action of the 5′−3′ ribonucleases XRN1 and XRN2^17^, ^18^ (Figure 2B). Among them, XRN1 is responsible for the degradation of cytoplasmic piRNA, and XRN2 is responsible for the degradation of nuclear piRNA. Upon dissociation from the PIWI complex, piRNAs are vulnerable to exonuclease degradation at both their 5′ and 3′ ends, and PIWI proteins protect piRNAs from degradation.^19^ In addition, the 3′‐terminal Nm of piRNA reduced its degradation via an exosome‐mediated decay pathway. piRNA sequences with G‐quadruplex structures can slow the rate of XRN1‐mediated degradation.^17^ In *Drosophila*, Nm also protects siRNA from complementarily dependent degradation. In contrast, pre‐piRNA pruning protects mouse piRNAs from degradation pathways that trigger complementarities. Deletion of pruning and Nm results in piRNA pathway blockade in mice^20^ (Figure 2C). Understanding the complex mechanisms of piRNA degradation helps elucidate the regulatory processes essential for spermatogenesis.

## 3 PIRNA FUNCTIONS AND RELATED MECHANISMS

Transposable elements (TE) expression in the animal germline is regulated by PIWI–piRNA complexes, which exert repressive effects mainly at transcriptional and post‐transcriptional levels.^21^ These PIWI proteins direct piRNAs to bind nascent RNAs through TE transcription sites and recruit the cofactors Panx, Nxf2 and Nxt1 to function in transcriptional TE silencing by forming complexes.^22^, ^23^, ^24^ In addition, piRNAs regulate protein‐coding genes and are associated with spermatogenesis. For example, MIWI and piRNA interact to activate the translation of many spermatogenic mRNAs.^25^ PIWI proteins in the mouse and *Drosophila* contribute to the stabilisation of mRNA in the germline by suppressing transposons and are associated with translational machinery.^26^, ^27^ It is believed that piRNAs modified by H3K9me3‐induced methylation can spread to neighbouring regions, leading to gene expression effects. PIWI–piRNA complexes exert epigenetic silencing by regulating its target genes through H3K9me3 and Pol II binding to the target genes^28^ (Figure 1). These discoveries broaden the scope of piRNA roles in controlling protein‐coding genes, yet the precise mechanisms underlying this regulation are still to be elucidated. Liu and colleagues found that the unique PIWI‐Ins module in PIWI clade proteins contributes to the determination of piRNA length.^29^ The longer piRNA has additional complementarities with the target mRNA, enhancing the assembly of the MIWI/eIF3f/HuR supercomplex and further promoting translational activation. Selective tuning of piRNA length by PIWI‐Ins modules is essential for spermatid development and male fertility.

Studies on piRNAs are gradually revealing their diverse biological functions, including affecting cell proliferation, migration, apoptosis, cell cycle and other activities.^30^ piRNA plays an important role as a biomarker in the diagnosis of acute myocardial infarction (AMI) and neuroblastoma (NB). In particular, hsa‐piR‐9010, hsa‐piR‐28646 and piRNA‐23619 were upregulated in AMI patients and piRNA‐MW557525 in NB patients. Further studies suggested that piRNA might play a role as a therapeutic target.^31^, ^32^ Hsa‐piR‐28059, hsa‐miR‐657 and hsa‐miR‐2110 were highly expressed in the stool samples of Autism spectrum disorder (ASD) patients. 84 piRNAs and 3 piRNAs‐targeting genes (CFLAR，GOLGA6L2 and SLC2A4) were up‐regulated, and they are involved in pathways related to the development of autism and are associated with intestinal permeability and inflammation.^33^ In intestinal tumours, piR‐823, piR‐020619, piR‐020450 and piRNA‐54265 levels in serum and tissues of patients with colorectal cancer are significantly increased, which could serve as key biomarkers and therapeutic targets in colorectal cancer management.^34^, ^35^, ^36^ piRNAs also play important roles in neuronal development, learning and memory. Aberrant tau expression in *Drosophila* brain reduces PIWI and piRNA levels and accelerates neuronal death due to TE disinhibition in neurodegenerative diseases.^37^, ^38^ In addition, the expression of some piRNAs is abnormal in Alzheimer's disease patients.^39^ piRNA plays a potentially important role in different tissues, such as liver, heart, retinal cells, retinal pigment epithelial cells and peripheral blood.^40^, ^41^, ^42^, ^43^, ^44^, ^45^, ^46^, ^47^, ^48^ These findings not only expand our understanding of the function of piRNAs but also provide new perspectives and possibilities for the study of related diseases. piRNA has high stability in serum and can maintain its integrity even under the influence of the external environment.^49^ Here, we collated the most recent studies of piRNAs as potential markers in related diseases (Table S1). It can be anticipated that piRNA has significant potential as a diagnostic marker for CVDs.

piRNAs exhibit variations in nomenclature across different databases. For instance, a piRNA denoted as ‘piR‐hsa‐237’ in the piRBase repository is identified as ‘hsa‐piR‐000001’ in the piRNABank database and ‘hsa‐piR‐1’ in the piRNAdb database. The nomenclature of piRNAs often reflects their associations with PIWI proteins and specific sequence characteristics within a particular organism. The naming conventions employed by piRBase and piRNAdb typically comprise the prefix ‘piR’ representing piRNAs, followed by a species‐specific abbreviation.^50^, ^51^ piRNA is a relatively new biological regulator, and establishing a unified naming system will help to promote the research of piRNA. At present, various piRNA online databases have been established, which provide researchers with a wealth of piRNA sequence, biological characteristics, expression patterns and target information. These resources contribute to the in‐depth study of piRNA functions and promote the development of life sciences and clinical applications (Table 1).

**TABLE 1:** piRNA online database information.

Database	URL	Function	References
piRDisease	http://www.PIWIrna2disease.org/index.php	A database to assist piRNA function studies, which provides a comprehensive annotation of piRNA sequences and contains potential information on piRNA targets and disease‐related piRNAs	^52^
piRNAQuest	http://bicresources.jcbose.ac.in/zhumur/pirnaquest/	It contains 28 species and provides piRNA annotations according to different locations	^53^
piRNA cluster database	http://www.smallrnagroup‐mainz.de/piRNAclusterDB.html	The only piRNA cluster database that provides comprehensive information on multiple species, tissues and developmental stages	^54^
piRTarBase	http://cosbi6.ee.ncku.edu.tw/piRTarBase/	It is the only piRNA cluster database that provides comprehensive information on multiple species, tissues and developmental stages, piRNA target database, and predicted binding sites of piRNA and miRNAs	^55^
piRNAbank	http://pirnabank.ibab.ac.in http://gtrnadb.ucsc.edu	One of the earliest and comprehensive databases, rich in human, rat and other species	^56^
piRNApredictor	http://59.79.168.90/piRNA/indexphp	piRNA sequence information was predicted	^57^
IsopiRBank	http://mcg.ustc.edu.cn/bsc/isopir/index.html	Prediction of piRNA sequence information, a comprehensive piRNA database, covering the sequence information, biological characteristics and expression patterns of piRNA	^50^

## 4 PIRNAS IN CVDS

In recent years, researchers have gradually recognised that ncRNAs play an important regulatory role in cardiovascular health and disease. This review will focus on the role of piRNA in CVDs, focusing on its impact on MI, HCM and other important diseases. To summarise the mechanisms of piRNA in CVDs development and provide new ideas for future treatment and prevention.

### 4.1 Myocardial infarction

**FIGURE 3:**
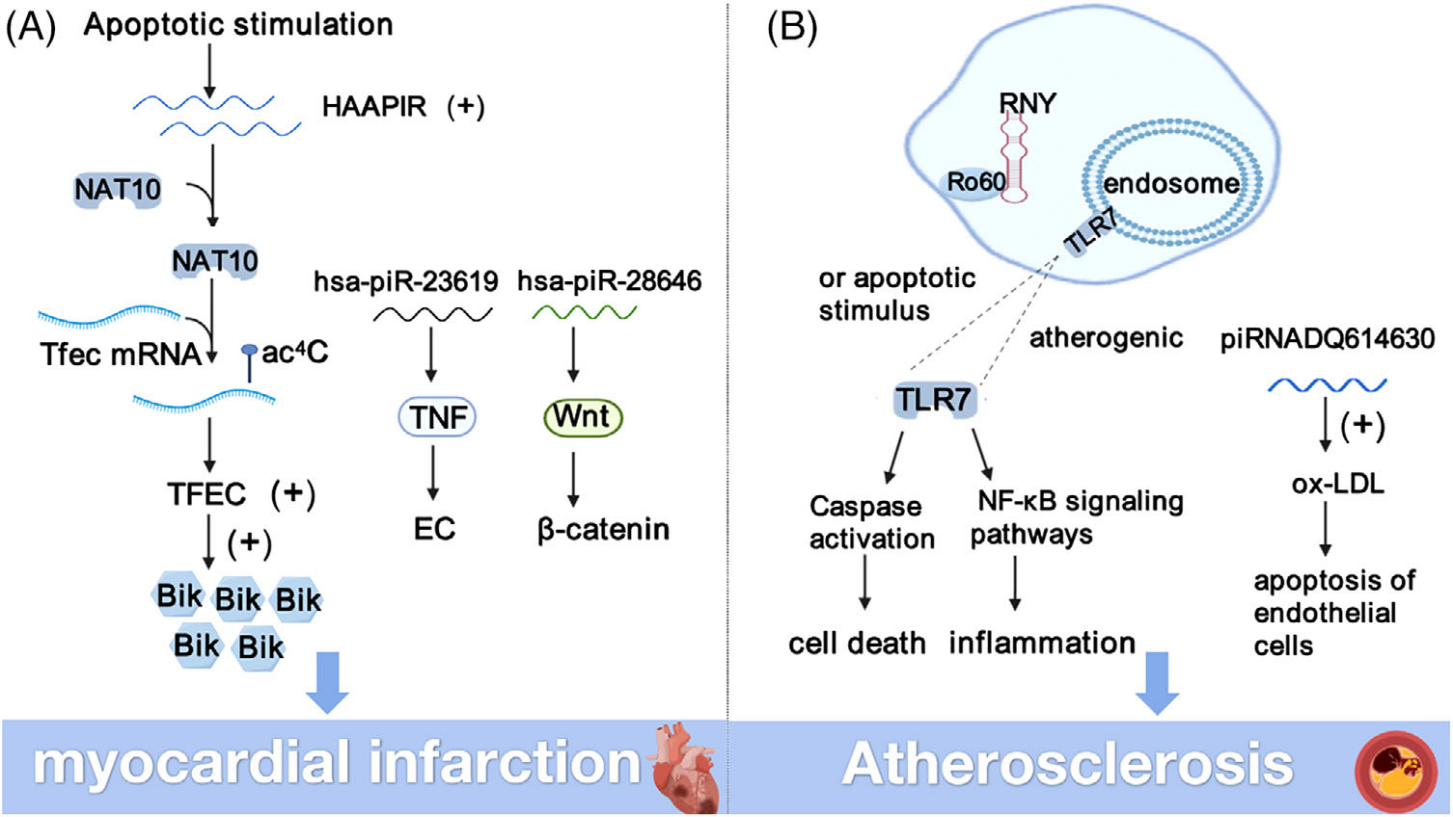
The mechanism of piRNA in myocardial infarction and atherosclerosis. (A) HAAPIR affects Tfec through targeted NAT10 mRNA ac^4^C acetylation, and Tfec can be further raised by Bik transcription and regulation of myocardial injury caused by cardiomyocyte apoptosis. And hsa‐piR‐23619 promotes inflammation, endothelial cells by TNF signalling pathways and hsa‐piR‐28646 activation of Wnt signalling pathways, affect the process of AMI. (B) YsRNA‐5p transcript promotes foam cell apoptosis and inflammation by binding to Ro60. Overexpression of piRNA‐DQ614630 can accelerate the ox‐LDL‐induced apoptosis of endothelial cells.

MI is cell death resulting from severe and sustained damage to cardiomyocytes. At present, serum biomarkers for the clinical diagnosis of MI include troponin, CK‐MB, etc.^31^, ^58^ However, these indicators are greatly affected by other factors, and new myocardial markers are urgently needed to replace them. Huang et al. first found changes in piRNA abundance and expression in the serum of patients with AMI, which has potential diagnostic significance. In this study, serum samples were collected from AMI patients and healthy patients. After RNA sequencing and bioinformatics analysis. It was observed that most piRNAs were upregulated. In particular, hsa‐piR‐9010, hsa‐piR‐28646 and hsa‐piR‐23619 showed marked elevation in the serum of patients suffering from AMI. In terms of mechanism, hsa‐piR‐23619 mainly acts on TNF signalling pathway to induce inflammatory response of endothelial cells (EC) after MI and affect its vascular function.^31^ In the EC of the infarct zone, hsa‐piR‐28646 activated the Wnt signalling pathway and accumulated β‐catenin, thereby affecting angiogenesis after MI^59^ (Figure 3A). piRNA may affect the process of AMI by regulating cardiomyocyte apoptosis, inflammatory response and angiogenesis. This suggests that piRNA could serve as a promising novel biomarker for the early detection of AMI.

Heart apoptosis‐associated piRNA (HAAPIR) is a proapoptotic factor with the ability to cause cardiomyocyte apoptosis and mitochondrial fragmentation, but does not affect myocardial necrosis.^11^, ^60^ HAAPIR interacts with NAT10 to enhance ac^4^C acetylation of Tfec mRNA transcripts and may be an effective potential therapeutic target for alleviating MI and ameliorating ischemia‐reperfusion injury (Figure 3A). By inhibiting HAAPIR, H_2_O_2_‐induced cardiomyocyte apoptosis and mitochondrial fragmentation could be prevented.^11^ Knockout of HAAPIR attenuated cardiomyocyte injury and inhibited cell apoptosis in vivo. This suggests that regulation of HAAPIR may play a key role in cardiac protection. There are also reports that the level of LINE‐1 was significantly increased in rat hearts with reperfusion injury. Activation of the Akt/PKB pathway can inhibit LINE‐1 expression and improve cardiac injury.^61^

### 4.2 Atherosclerosis

Atherosclerosis (AS) is a common CVD characterised by monocyte aggregation, foam cell formation, lipid deposition and smooth muscle cell proliferation.^62^ These pathological changes eventually lead to intimal hyperplasia, plaque rupture and thrombosis. MAPK, TLR, ROS and other signalling pathways participate in the process of AS.^63^ Ox‐LDL‐induced EC apoptosis, inflammation and oxidative stress are key risk factors for AS. Wang et al. treated rat ECs with bovine LDL to mimic EC injury. piRNA‐DQ614630 overexpression accelerated ox‐LDL‐induced apoptosis in ECs as determined by RNA high‐throughput sequencing and qPCR^64^ (Figure 3B). Some groups also believe that YRNA and YsRNA fragments blocked by extracellular vesicles (EVs) are significant in the development of AS.^65^ YRNA, potentially originating from miRNA and piRNA, primarily influences AS progression by modulating the binding sites of various proteins, such as nucleotide, Ro60, La, hnRNPK, hnRNPI. YsRNA‐5p transcript facilitates apoptosis in foam cells and triggers an inflammatory response through its interaction with the Ro60 protein. It can also activate the monocyte/macrophage death pathway and inflammatory response by enhancing caspase‐dependent and NF‐κB‐dependent pathways. In addition, YsRNA is highly expressed in the blood of patients with AS.^65^ The transcript product of YsRNA‐5p was positively correlated with high levels of high‐sensitivity C‐reactive protein (hs‐CRP), as well as proatherogenic proteins ApoB and low‐density lipoprotein (LDL), and negatively correlated with levels of the atherosclerotic lipoprotein ApoA‐I. YsRNA may be used as a potential biomarker to monitor the pathogenesis of AS (Figure 3B).

The gut microbiota also plays an important role in the progression of AS, contributing to the development of AS by reducing butyrate through the production of intermediate metabolites such as trimethylamine N‐oxide (TMAO), LPS, and phenylacetylglutamine (PAGln).^66^, ^67^ AS is a complex CVD, and its pathogenesis involves multiple levels of regulatory networks, including cell signalling pathways, RNA regulation, and the intervention of gut microbes. Given the important relationship between piRNAs and gut microbiota, it can be predicted that piRNAs may become potential therapeutic targets for AS.

### 4.3 Hypertrophic cardiomyopathy

**FIGURE 4:**
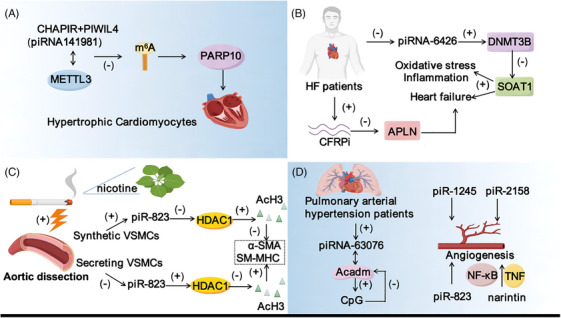
Mechanisms of piRNA in different CVDs. (A) The combination of CHAPIR and PIWIL4 affects the expression of PARP10 by targeting METTL3‐mediated m^6^A modification, which is involved in HCM. (B) piRNA‐6426 protects against hypoxia‐induced cardiomyocyte dysfunction and heart failure by promoting DNMT3B‐mediated SOAT1 methylation and CFRPi by inhibiting APLN promote cardiac fibrosis leads to HF. (C) piRNA‐823 affects VSMC function through HDAC1 in aortic dissection, in which nicotine in tobacco stimulates AD. (D) piRNA‐63076 is involved in hypoxia‐induced PASMC proliferation through Acadm in pulmonary hypertension. piRNA also plays a role in angiogenesis. HCM, hypertrophic cardiomyopathy; HF, heart failure; AD, aortic dissection.

Long‐term HCM can easily lead to HF.^68^ Studies have found that piRNAs are associated with HCM, and it may emerge as a novel serum biomarker for diagnosing HCM. NAB1 and NAB2 are transcription factors that can bind to EGR1. Overexpression of NAB1 can inhibit pressure overload‐induced HCM in response to cardiac pathological stimulation.^69^ piR‐141981 (CHAPIR) is highly expressed in hypertrophic cardiomyocytes.^70^ CHAPIR inhibited the m^6^A methylation of PARP10 mRNA by blocking METTL3‐mediated RNA methylation activity, resulting in METTLL3‐YTHDF2‐dependent degradation of PARP10 mRNA transcripts and increased PARP10 expression, leading to enhanced nuclear accumulation and NFATC4 activity (Figure 4A).

CHAPIR deficiency attenuates HCM and restores cardiac function. In summary, m^6^A methylation may contribute to HCM development by regulating kinase activity and intracellular signalling pathways Akt and ERK1/2, MAPK, etc.^71^ The increased expression of m^6^A RNA methylase METTL3 promotes HCM but does not affect cardiac function.^72^, ^73^ piRNA expression and abundance change during HCM.^74^ Overall, this study provides the first genome‐wide analysis of many piRNAs in the cardiac system and provides new insights into the molecular etiology of piRNAs and retrotransposons in the heart.

### 4.4 Heart failure

HF is the final stage of the development of CVDs. Persistent hypoxia and ischemia in cardiomyocytes caused by pressure overload and myocardial blockage are the primary contributors to the development of HF and even death.^75^ After cardiomyocyte injury, glucose absorption dysfunction, mitochondrial damage, enhanced ROS accumulation, and aggravated inflammatory response are observed.^76^ In the mouse model of HF, DNMT3B protein, a pivotal DNA methyltransferase in cardiac tissue, exhibits significant suppression.^77^, ^78^ DNA methyltransferases are pivotal in the pathogenesis of CVDs.^79^ piRNA‐6426 levels in the blood of HF patients presented lower compared to the control group. To investigate the role of piRNA‐6426 in cardiomyocyte functionality, the HF cell model was created through the induction of hypoxia in rat cardiomyocytes. Interestingly, it was observed that overexpression of piRNA‐6426 significantly improved cardiac function in rats with HF. At the mechanistic level, piRNA‐6426 interacts with DNMT3B, and it promotes the methylation of the SOAT1 promoter by recruiting DNMT3B, thereby inhibiting apoptosis, inflammation and oxidative stress of cardiomyocytes, and further affecting the progression of HF. This finding suggests that piRNA‐6426 is expected to be a new potential target for the diagnosis and treatment of HF^80^ (Figure 4B). This provides useful clues for further research into the treatment of HF in the future. Furthermore, piRNA‐000691 (CFRPi) promotes the development of myocardial fibrosis by targeting APLN. By knocking CFRPi, it can significantly reduce myocardial fibrosis and improve the systolic and diastolic function of the heart, which has the potential to be a new target for the treatment of HF^47^ (Figure 4B).

Yang and colleagues have achieved remarkable results in the isolation of serum exosomes from HF patients and healthy volunteers.^81^ They successfully extracted RNA from exosomes and performed RNA sequencing and bioinformatics analysis. Hsa‐piR‐020009 and hsa‐piR‐006426 are significantly downregulated in patients with HF. Notably, serum exosomes from HF patients exhibited distinct piRNA expression profiles. This result indicates that piRNA holds promise as a biomarker for HF, offering a novel avenue for its future diagnosis and therapeutic strategies.

### 4.5 Aortic dissection

Aortic dissection (AD), a severe cardiovascular condition, exhibits significant lethality. It is characterised by the tear of the aortic intima with sudden and severe pain. Factors such as smoking, hypertension, age and gender have been shown to influence the onset of AD.^82^ Li et al. found a high expression level of piR‐823 in AD patients and mice and a significant correlation with the disease. piR‐823 has been confirmed to promote the proliferation, migration and phenotypic transformation of vascular smooth muscle cells (VSMCS). This effect may be achieved through the mechanism of H3K9ac and H3K27ac regulating the acetylation of histone 3 (H3), and then directly binding to inhibit the expression of HDAC1^83^ (Figure 4C). Studies have also found that nicotine can induce the occurrence of AD, which provides a new therapeutic target for clinical prevention and treatment of AD. The critical role of piR‐823 in the regulation of VSMCs and AD is highlighted.

### 4.6 Pulmonary arterial hypertension

Increased pulmonary artery pressure can induce vascular stiffness, resulting in stenosis and impaired right ventricular function, significantly threatening human health.^84^, ^85^ The Lipps research team's comparative analysis of extracellular vesicle‐derived piRNAs from pulmonary hypertension patients and healthy subjects established their potential as biomarkers indicative of disease severity. In addition, upregulation of piRNA‐DQ593039, which was associated with pulmonary hypertension, was also demonstrated in the study.^70^ In pulmonary artery smooth muscle cells (PASMCs), increased expression of piR‐63076 induces degradation of acadm by promoting the methylation and transcriptional activity of adjacent CpG islands, leading to an increase in piRNA in pulmonary vessels^86^ (Figure 4D). This study raises the possibility that piRNA may serve as a novel biomarker for pulmonary hypertension and a potential target for clinical treatment.

### 4.7 Angiogenesis

Vascular endothelial cell proliferation, migration and capillary bud formation are key events in angiogenesis.^87^ piRNA‐specific inhibitors can reduce the expression level of vascular endothelial growth factor (VEGF) in EC, thereby affecting the formation of new blood vessels. The concentration of piRNA‐823 is positively correlated with the levels of VEGF and IL‐6 in extracellular vesicles of vascular ECs^88^ (Figure 4D). By regulating the expression of piRNA in Human Umbilical Vein Endothelial Cells (HUVECs), narintin can improve the angiogenesis ability of HUVECs through the NF‐κB and TNF signal transduction pathways and promote the healing of bone defects^89^, ^90^, ^91^ (Figure 4D). The findings imply that piRNA may exert a crucial regulatory function in the processes of angiogenesis and bone defect healing.

The expression of piR‐1245 was significantly enhanced in human retinal endothelial cells (HRECs) under hypoxic condition. piR‐1245 knockdown decreased new blood vessel formation, inflammation protein levels in HRECs, and retinal apoptotic cell numbers. Upon further analysis, the JAK2/STAT3 pathway was found to be inhibited, and the expression of hypoxia‐inducible factor‐1α and VEGF was also reduced. These results reveal that piR‐1245 is involved in the regulation of retinal angiogenesis by regulating the expression of HIF‐1α and VEGF under hypoxic conditions^43^ (Figure 4D). piR‐2158 is involved in Wnt and IL11 signalling pathways to inhibit angiogenesis in breast cancer.^92^ Many inhibitors of Wnt regulation exhibit proangiogenic effects. In the ECs of the infarct zone, β‐catenin accumulation activates Wnt signalling, enhancing capillary density in the MI scar zone when inhibited. Stabilising NP12 of β‐catenin by inhibition of GSK3β can activate Wnt signalling, thereby promoting angiogenesis^93^ (Figure 4D). In addition, Wnt‐1‐inducible secretory protein‐1 (WISP‐1) could further promote cardiac angiogenesis after MI through regulating histone deacetylases.^94^ This finding provides new theoretical support for the use of Wnt signalling to regulate angiogenesis and improve cardiac function in MI recovery.^95^

### 4.8 Myocardial fibrosis

Myocardial fibrosis is a process of cardiac remodelling caused by myofibroblast invasion and collagen secretion.^96^ Research indicates the Wnt/β‐catenin pathway crucially governs cardiac fibrosis post‐MI. Studies have shown that aldehyde dehydrogenase‐2 (ALDH2) activity may be regulated by GSK3β‐, Wnt‐1‐ and WISP‐1‐mediated downregulation of β‐catenin in cardiac fibroblasts, resulting in reduced cardiac fibrosis and improving cardiac function and inhibiting fibroblast proliferation in a pressure overload mouse model.^95^ In parallel, transfection with miR‐154 inhibitor also reduced the expression of β‐catenin and myocyte proliferation by interacting with DKK2.^97^ During *T. cruzi* infection, the AP‐1 protein FOS can play a role in early pathogenesis by activating fibre genes and piRNA expression increases.^98^ It is worth noting that more studies have not yet shown that there is a direct functional link between myocardial fibrosis and piRNA, which provides a new direction for future research.

Researchers identified a heart‐enriched piRNA‐000691 that is specifically expressed in cardiac fibroblasts. The piRNA expression in the progress of HF, and in vitro and in vivo experiments confirmed that can promote cardiac fibrosis. Knockout of piRNA‐000691 reduced cardiac fibrosis and improved cardiac function in mice. Mechanistically, piRNA‐000691 activated cardiac fibroblasts by inhibiting APLN, while knockdown of APLN exacerbated fibrosis. In addition, Pi3k‐AKT‐mTOR signalling pathway was the downstream pathway of piRNA‐000691 affecting APLN. These findings suggest that targeting piRNA‐000691 may reduce fibrosis, restore cardiac function and have the potential to prevent HF by increasing APLN expression.^47^

### 4.9 Others

Congenital heart defect (CHD), a prevalent birth defect in newborns, refers to structural or functional abnormalities of the heart and large blood vessels that occur during fetal development and may include cardiac ventricular wall between holes, abnormal blood vessels connection or heart valves, and associated with ROBO4, Hnrnpa1 variation.^99^, ^100^ The relationship between CHD and piRNA is becoming an interesting topic in the field of cardiac development and disease research. In pregnant women's exosomes, two distinct piRNAs, hsa‐piR‐009228 and hsa‐piR‐016659, show increased expression, serving as noninvasive prenatal diagnostic markers for the detection of fetal congenital malformations.^101^ Dilated cardiomyopathy (DCM) manifests as a condition marked by irregular expansion of the ventricles and impaired systolic activity. DCM may limit the effective pumping of blood by the heart, causing ventricular enlargement and HF.^102^ It can be caused by a variety of factors, including viral infections, autoimmune diseases, and drug side effects. The study found that lncRNA CFIRL rises significantly in the DCM hearts, and its knockout reduces cardiac dysfunction and fibrosis. CFIRL activates IL‐6 by recruiting ENO1 and promotes cardiac hypertrophy. Targeting CFIRL is expected to be a new strategy to prevent cardiac remodelling in DCM. Although there is currently no direct evidence to definitively link piRNA to the pathogenesis of DCM, we speculate that piRNA can be expressed differently in patients with HF by targeting relevant symptoms, such as piRNA‐6426, hsa‐piR‐020009, and hsa‐piR‐006426. It may provide indirect therapeutic strategies for DCM.^80^, ^81^ Future studies may reveal the specific mechanisms of piRNA in CHD and DCM and provide clues for the development of new diagnostic and therapeutic approaches.

**FIGURE 5:**
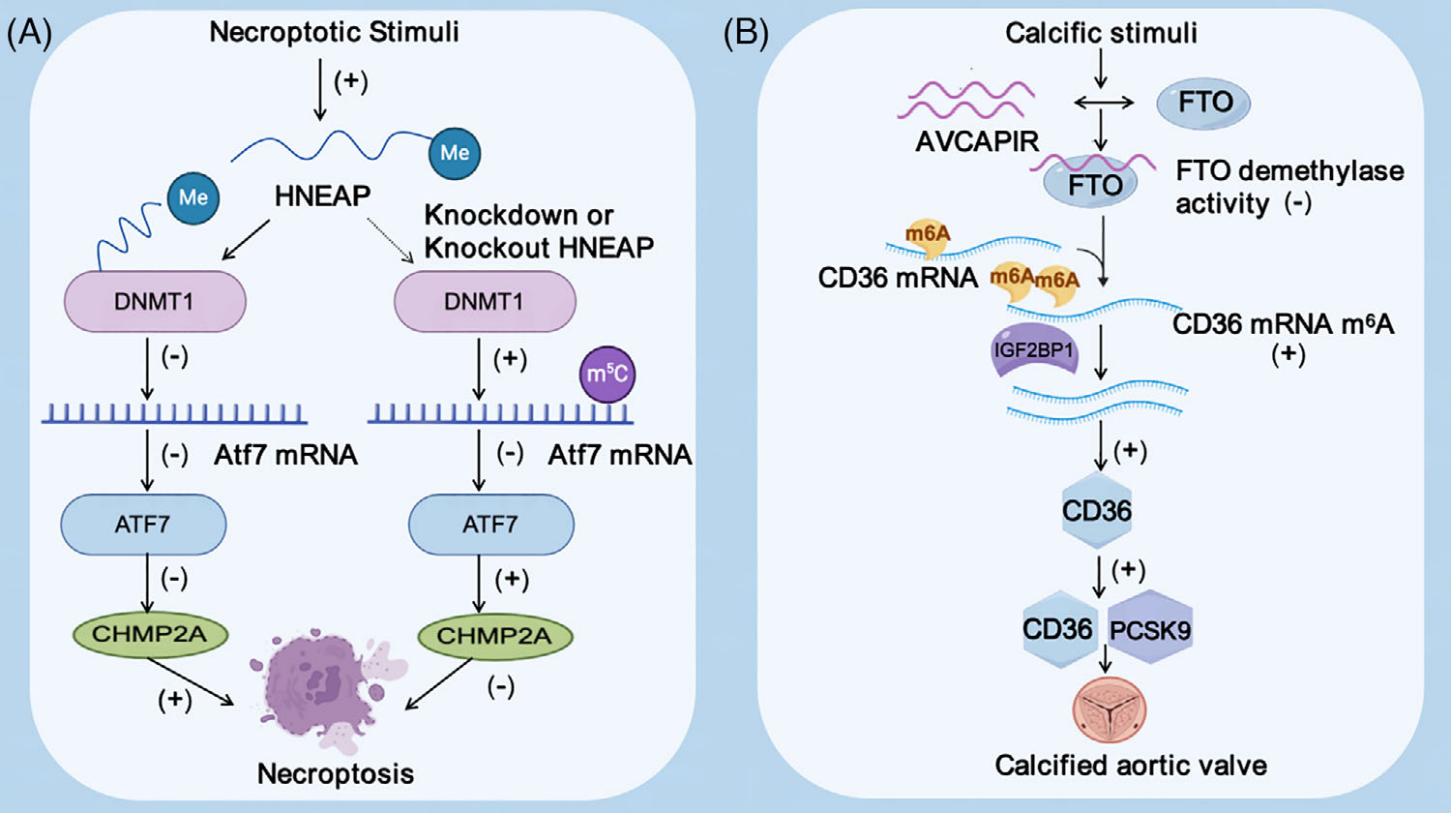
Regulation of cardiac injury and valve calcification. (A) HNEAP influences the m^5^C methylation of Atf7 mRNA through interaction with DNMT1, thereby regulating the cardiac damage caused by necrosis in ischemic heart disease. (B) AVCAPIR blocked the demethylation of CD36 mRNA by inhibiting the demethylase activity of FTO to enhance its stability and further stabilised CD36 protein by IGF2BP1, promoting the stability of PCSK9 and accelerating the process of aortic valve calcification.

HNEAP, linked to cardiac necrosis piRNA, enhances Atf7 expression via its interaction with DNMT1, thereby diminishing the presence of necrosis inhibitors like Chmp2a, which leads to cardiomyocyte necrosis. Reveals the HNEAP‐DNMT1‐ATF7‐CHMP2A shaft in the regulation function of cardiomyocyte necrosis^103^ (Figure 5A). Meanwhile, AVCAPIR binds the m^6^A demethylase FTO and inhibits its activity, resulting in the hypermethylation of CD36 mRNA downstream of FTO. Subsequently, the intervention of m^6^A reading protein IGF2BP1 promoted the stability and expression level of CD36 RNA. The upregulation of CD36 at protein level is not only related to valve calcification but also related to the stability and expression level of PCSK9 protein that promotes valve calcification. This eventually leads to aortic valve calcification^104^ (Figure 5B).

Diabetes not only is an important participant in the development of CVDs and is closely related to islet hormone secretion. Insulin resistance is characterised by decreased insulin‐mediated glucose utilisation, resulting in decreased sensitivity and responsiveness to insulin, and the inability to maintain a steady state of blood glucose. Studies have shown a link between RNA and metabolic phenotypes, with piRNA‐48383 associated with Insulin resistance, but its role in CVDs needs to be further investigated.^105^ Expression of PIWL2 and PIWIL4 genes, as well as piRNA, was found in rat islet cells by microarray technology, and the silencing of these genes leads to decreased insulin secretion and enhances the resistance of islet cells to cytokine‐induced cell death. In addition, also found in the diabetic rat islet cells, piRNA‐DQ732700 and piRNA‐DQ746748 high expression and the shortcomings of glucose induced insulin release.^106^

## 5 OUTLOOK AND DISCUSSION

At present, strategies for the treatment of CVDs mainly focus on controlling risk factors, preventing disease progression and implementing personalised treatment. In this context, ncRNA has become a hot spot in medical research and has also shown its great potential regulatory function in CVD. The Nm modification at the 3′ end is a prominent feature of piRNA, which can be verified by the RTL‐P method.^107^, ^108^ Specifically, reverse transcription was performed with anchored or unanchored RT primers at low concentrations of deoxyribonucleoside triphosphate (dNTP), followed by PCR. The presence of Nm‐modified piRNA at the 3′ end can be detected under a low concentration of dNTP. The length of tsRNAs is similar to piRNAs, and some tsRNAs can also interact with PIWI proteins to play a variety of biological functions.^109^ Zhang and colleagues found that td‐piR (Glu) forms a complex with PIWIL4 and acts as a key signalling molecule to regulate membrane protein expression.^13^ However, it may be a tsRNA rather than a piRNA. Xiang et al. examined the expression of PIWI protein in cardiac tissue.^110^ Rajan et al. demonstrated that many piRNAs exist in the cardiac system, and specific piRNAs in serum have the potential to target and predict MI.^74^ Most piRNAs expressed in CVDs rely on PIWI proteins to exert their unique biological functions, such as CHAPIR and PIWIL4, AVCAPIR and PIWIL1.^72^ There are no reports that piRNA regulates CVDs via a PIWI‐independent mechanism. However, some PIWI proteins have been found to function through piRNA‐independent mechanisms. PIWIL1 can bind to UPF1 protein molecules and mediate nonsense mRNA decay (NMD).^111^ This suggests to us that PIWI‐independent piRNAs may have unknown special functions. Exploring the special functions of piRNAs in CVDs and the molecular regulatory mechanism behind piRNAs independent of the PIWI protein will provide a new perspective for the precise targeted therapy of CVDs.

**FIGURE 6:**
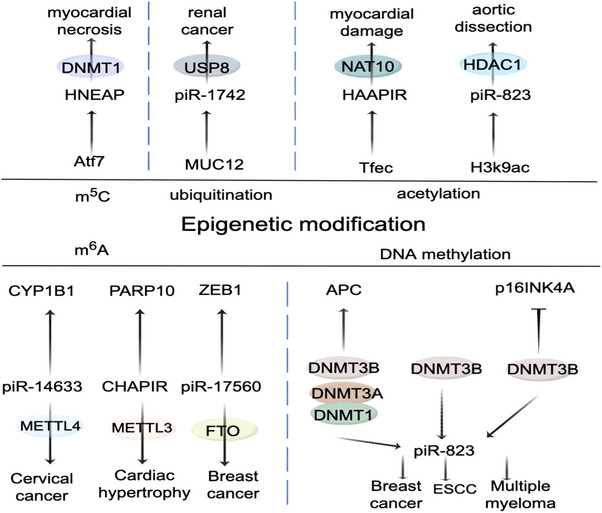
Epigenetic modifications of piRNAs in different diseases. In this figure, the bottom is the type of disease triggered, the middle half is the enzyme mediating the modification and the associated piRNA, and the top is the target of the final effect. The main types of epigenetic modifications involved are m^6^A, m^5^C, DNA methylation, ubiquitination and acetylation.

Drugs, cardiac assist devices and heart transplantation are the main options for the clinical treatment of CVDs. Cardiac assist devices treat diseases by improving the patient's haemodynamics. However, there is a higher risk of complications, such as embolism and infection. Heart transplantation is an effective treatment for patients with end‐stage HF, but the problem of donor shortage and immune rejection seriously limits the treatment of patients. Long‐term use of traditional cardiovascular drugs can lead to potential gastrointestinal reactions and liver and kidney damage. Therefore, there is an urgent need to develop new drugs for the treatment of CVDs. ox‐LDL can accelerate the progression of AS by promoting the expression of individual piRNAs, leading to EC apoptosis.^64^ In addition, piRNAs are associated with pathways and networks related to EC senescence and cell cycle.^112^ Revealing the important role and potential therapeutic value of piRNA will provide theoretical support for drug target research in the cardiovascular field. However, more experimental studies and clinical trials are still needed to verify the feasibility, efficacy and drug availability of piRNAs as therapeutic targets for CVDs. Delivery of therapeutic piRNA drugs to the cardiovascular system is a potential targeted therapeutic strategy for the treatment of CVD. Nanotechnology is expected to become an important means to assist piRNA in the treatment of CVD. Lipid nanoparticles (LNP) are clinically approved nonviral nucleic acid delivery systems, which can effectively carry out nucleic acid encapsulation, cell delivery and endosomal release. The LNP encapsulation system can be used to transport nucleic acid molecules such as piRNA or piRNA interference through tissues to vivo and has low cytotoxicity and immunogenicity. Cell membrane‐coated nanoparticles (CMCNs) are an emerging class of nanocarriers, which can target damaged myocardial tissue for nucleic acid delivery by using biological membrane‐coated nanocomposites.^113^ In addition, injectable hydrogels as carriers can effectively prolong the retention of nucleic acids at the infarct site, thereby improving cardiac function.^114^ Admittedly, there are significant limitations in pharmacological treatment of inherited heart disease, and gene editing technology may be a potential solution to repair or improve the genetic defects of patients. CRISPR/Cas9 gene editing technology can correct or mutate genes to restore the normal physiological function of abnormal cardiomyocytes, which provides a new idea for the treatment of CVDs. Some technical challenges and ethical issues are the main obstacles on the way to its wide application. piRNA‐mediated gene silencing is the main molecular mechanism of piRNA regulating CVD.^9^ piRNA searches for target molecules through complementary base pairing, while PIWI proteins are responsible for cleaving the target RNA and degrading it.^115^, ^116^ Compared to other similar molecular targets (such as miRNAs), Li et al. found that PIWI/piRNA complexes have better targeting and cleavage ability towards target genes.^117^ The endogenous gene silencing completed by piRNA can be inherited across generations, and this piRNA‐mediated interference (piRNAi) is more effective than RNA interference (RNAi).^118^ It also brings new insights and theoretical possibilities for the treatment of CVD by regulating the expression of protein‐coding genes through piRNA. In addition, PIWI–piRNA complexes can recruit other proteins to regulate gene expression through epigenetic means, affecting the progression of various human diseases (Figure 6).

The exploration of piRNA in the cardiovascular system is at an early stage compared to studies of other diseases, but we cannot deny its important value in clinical applications. In addition, piRNA expression is highly specific in samples such as body fluids and serum. The specificity and sensitivity of some piRNAs (piR‐020619 and piR‐020450) have exceeded those of current clinical tumour markers (CEA and CA‐199).^35^ For potential CVD patients, detection of their serum piRNA levels may be a convenient and accurate diagnostic method in the future. In summary, piRNA studies not only broaden our knowledge of CVDs treatment but also play an important role in the diagnosis and treatment of CVDs. However, these novel piRNA‐dependent CVD protocols need further validation and improvement to solve the possible new problems that may arise.

## AUTHOR CONTRIBUTIONS

All authors provided direction and guidance throughout the preparation of this manuscript. Bo Li, Kai Wang and Wei Cheng drafted the manuscript. Bo Fang and Ying Hui Li assisted in drafting the manuscript and design of ideas. Bo Li, Kai Wang and Wei Cheng contributed equally to this work. Su Min Yang, Mei Hua Zhang, Yun Hong Wang and Kun Wang reviewed and made significant revisions to the manuscript. All authors have read and approved the final manuscript.

## CONFLICT OF INTEREST STATEMENT

The authors declare that they have no competing interests.

## ETHICS STATEMENT

Not applicable.

## Supporting information

Supporting Information
