# Supplementary material for: Recent advances of PIWI‐interacting RNA in cardiovascular diseases
Source: Clin Transl Med. 2024 Jul 31;14(8):e1770. doi: 10.1002/ctm2.1770 (PMC11290350; doi:10.1002/ctm2.1770)
Supplement: Supplementary file 1 — Supporting Information [file CTM2-14-e1770-s001.docx]

**Supplementary Table S1. piRNAs as potential biomarkers in related diseases.**

| piRNAs name | Diseases | Samples | Expression | References |
| --- | --- | --- | --- | --- |
| piRPG (PPP1R12B) | Abdominal aortic aneurysm | Aneurysm specimens | Down-regulation | ^1^ |
| piR-004153, piR001918, piR-020401 | Endometriosis | Sample of saliva | Down-regulation | ^2^ |
| piR-127, piR-2106 | Gastrointestinal cancer | Blood sample and muscle biopsy | Down-regulation | ^3^ |
| piR-5937, piR-28876 | Colorectal cancer | Serum | Down-regulation | ^4^ |
| piR-54265 |  | Serum | Up-regulation | ^5^ |
| piR-15254, piR-1029, novel-piR-35395, novel-piR-32132 and novel-piR-43597 | Hepatocellular carcinoma (HCC) | Exosomes isolated from serum | Up-regulation | ^6^ |
| piR-13643, piR-21238 | Papillary thyroid carcinoma (PTC) | PTC samples | Up-regulation | ^7^ |
| piRNA-MW557525 | Neuroblastoma | NB organization | Down-regulation | ^8^ |
| has-piR-019914, hsa-piR-020450 | Myelodysplastic (MDS) | Serum | Up-regulation | ^9^ |
| piR-4987, piR-20365, piR-20485, piR-20582 | Breast cancer | Tumor tissue | Up-regulation | ^10^ |
| hsa-piR-9010, hsa-piR-28646, hsa-piR-23619 | Acute myocardial infarction | Serum | Up-regulation | ^11^ |
| hsa-piR-164586 | Non-small cell lung cancer | Exosomes isolated from serum | Up-regulation | ^12^ |
| hsa-piR-27620, hsa-piR-27124 | Rheumatoid arthritis (RA) | RNA was extracted from blood samples | Up-regulation | ^13^ |

**Abbreviations.**

| Acute myocardial infarction | AMI |
| --- | --- |
| Aldehyde dehydrogenase-2 | ALDH2 |
| Argonaute | Ago |
| Atherosclerosis | AS |
| Autism spectrum disorder | ASD |
| Cardiovascular diseases | CVDs |
| Cell membrane-coated nanoparticles | CMCNs |
| DNA methyltransferase 1 | DNMT1 |
| Endothelial cells | ECs |
| Heart failure | HF |
| High-sensitivity C-reactive protein | hs-CRP |
| Histone 3 | H3 |
| HUA ENHANCER 1 | HEN1 |
| Human retinal endothelial cells | HRECs |
| Human Umbilical Vein Endothelial Cells | HUVECs |
| Hypertrophic cardiomyopathy | HCM |
| Lipid nanoparticles | LNP |
| Low-density lipoprotein | LDL |
| Myocardial infarction | MI |
| N4-acetylcytidine | ac^4^C |
| N6-methyladenosine | m^6^A |
| Neuroblastoma | NB |
| Non-coding RNAs | ncRNAs |
| Obesity-associated protein | FTO |
| Phenylacetylglutamine | PAGln |
| PIWI-interacting RNA | piRNA |
| Pulmonary artery smooth muscle cells | PASMCs |
| Transposable element | TE |
| Trimethylamine N-oxide | TMAO |
| Vascular endothelial growth factor | VEGF |
| Vascular smooth muscle cells | VSMCS |
| Wnt-1-inducible secretory protein-1 | WISP-1 |
| Zucchini | Zuc |
| 2’-O-methylation | Nm |
| 5-Methylcytidine | m^5^C |

**References:**

1 Jia, D. *et al.* Revealing PPP1R12B and COL1A1 as piRNA pathway genes contributing to abdominal aortic aneurysm through integrated analysis and experimental validation. *Gene* **897**, 148068 (2024). <https://doi.org/10.1016/j.gene.2023.148068>

2 Dabi, Y. *et al.* New class of RNA biomarker for endometriosis diagnosis: The potential of salivary piRNA expression. *Eur J Obstet Gynecol Reprod Biol* **291**, 88-95 (2023). <https://doi.org/10.1016/j.ejogrb.2023.10.015>

3 Molfino, A. *et al.* Small non-coding RNA profiling in patients with gastrointestinal cancer. *J Cachexia Sarcopenia Muscle* **14**, 2692-2702 (2023). <https://doi.org/10.1002/jcsm.13343>

4 Vychytilova-Faltejskova, P. *et al.* Circulating PIWI-Interacting RNAs piR-5937 and piR-28876 Are Promising Diagnostic Biomarkers of Colon Cancer. *Cancer Epidemiol Biomarkers Prev* **27**, 1019-1028 (2018). <https://doi.org/10.1158/1055-9965.EPI-18-0318>

5 Mai, D. *et al.* Serum piRNA-54265 is a New Biomarker for early detection and clinical surveillance of Human Colorectal Cancer. *Theranostics* **10**, 8468-8478 (2020). <https://doi.org/10.7150/thno.46241>

6 Rui, T. *et al.* Serum Exosome-Derived piRNAs Could Be Promising Biomarkers for HCC Diagnosis. *Int J Nanomedicine* **18**, 1989-2001 (2023). <https://doi.org/10.2147/IJN.S398462>

7 Chang, Z. *et al.* PIWI-interacting RNAs piR-13643 and piR-21238 are promising diagnostic biomarkers of papillary thyroid carcinoma. *Aging (Albany NY)* **12**, 9292-9310 (2020). <https://doi.org/10.18632/aging.103206>

8 Mi, T. *et al.* Activation of the p53 signaling pathway by piRNA-MW557525 overexpression induces a G0/G1 phase arrest thus inhibiting neuroblastoma growth. *Eur J Med Res* **28**, 503 (2023). <https://doi.org/10.1186/s40001-023-01493-w>

9 Georgoulis, V., Koumpis, E. & Hatzimichael, E. The Role of Non-Coding RNAs in Myelodysplastic Neoplasms. *Cancers (Basel)* **15** (2023). <https://doi.org/10.3390/cancers15194810>

10 Huang, G. *et al.* Altered expression of piRNAs and their relation with clinicopathologic features of breast cancer. *Clin Transl Oncol* **15**, 563-568 (2013). <https://doi.org/10.1007/s12094-012-0966-0>

11 Huang, Y. *et al.* Expression and diagnostic value of PIWI-interacting RNA by serum in acute myocardial infarction. *J Cardiol* **82**, 441-447 (2023). <https://doi.org/10.1016/j.jjcc.2023.06.015>

12 Li, Y. *et al.* Serum-derived piR-hsa-164586 of extracellular vesicles as a novel biomarker for early diagnosis of non-small cell lung cancer. *Front Oncol* **12**, 850363 (2022). <https://doi.org/10.3389/fonc.2022.850363>

13 Ren, R., Tan, H., Huang, Z., Wang, Y. & Yang, B. Differential expression and correlation of immunoregulation related piRNA in rheumatoid arthritis. *Front Immunol* **14**, 1175924 (2023). <https://doi.org/10.3389/fimmu.2023.1175924>
